# Healthy eating patterns associated with reduced risk of inflammatory bowel disease by lowering low-grade inflammation: evidence from a large prospective cohort study

**DOI:** 10.1186/s12916-024-03809-x

**Published:** 2024-12-18

**Authors:** Bin Xia, Yan Li, Linmin Hu, Peng Xie, Ningning Mi, Liyuan Lv, Zixin Liang, Yuxuan Sun, Ying Li, Xiaodong Jiang, Guinan Liu, Yuanyuan Feng, Yingxin Zhu, Bo Zhan, Qiangsheng He, Pingguang Lei, Jian Qi, Pengpeng Wang, Jinqiu Yuan

**Affiliations:** 1 Department of Epidemiology and Biostatistics, Clinical Big Data Research Center, The Seventh Affiliated Hospital, Sun Yat-Sen University, Shenzhen, Guangdong 518107 China; 2 Chinese Health Risk Management Collaboration (CHRIMAC), Shenzhen, Guangdong China; 3 School of Medicine, Sun Yat-Sen University, Shenzhen, Guangdong China; 4 School of Public Health (Shenzhen), Sun Yat-Sen University, Shenzhen, China; 5 Guangdong Provincial Key Laboratory of Gastroenterology, Center for Digestive Disease, The Seventh Affiliated Hospital, Sun Yat-Sen University, Shenzhen, Guangdong 518107 China; 6 The First School of Clinical Medicine, Lanzhou University, Lanzhou, Gansu 730000 China; 7 Department of Gastroenterology, Shenzhen Bao’an District Songgang People’s Hospital, No.2, Shajiang Road, Baoan District, , Shenzhen, Guangdong 518105 China; 8 Department of Occupational and Environmental Health, College of Public Health, Zhengzhou University, Zhengzhou, 450001 China

**Keywords:** Healthy dietary patterns, Crohn’s disease, Ulcerative colitis, Low-grade inflammation score

## Abstract

**Background:**

Limited epidemiological evidence exists regarding the role of healthy eating patterns in reducing the risk of Crohn’s disease (CD) and ulcerative colitis (UC). This study aimed to investigate the association between adherence to four established healthy eating patterns and subsequent CD or UC risk, and further examined whether these associations are linked to anti-inflammatory mechanisms.

**Methods:**

We conducted a prospective cohort study of 197,391 participants from the UK Biobank who completed at least one dietary questionnaire and were free from inflammatory bowel disease or cancer at baseline. Four dietary patterns were assessed, including Alternate Mediterranean Diet (AMED), Healthy Eating Index 2015 (HEI-2015), Healthful Plant-based Diet Index (HPDI), and EAT-Lancet. Cox proportional models with restricted cubic splines were applied to explore the associations. The potential role of low-grade inflammation in these associations was examined through mediation analysis.

**Results:**

During 2,193,436 person-years follow-up, 260 CD and 601 UC cases were identified. Higher AMED and HEI-2015 scores were associated with a reduced risk of CD but no UC, with no evidence against nonlinearity. These associations remained consistent across multiple sensitive and subgroup analyses. For dietary components, the fruits and monounsaturated fatty acids: saturated fatty acids ratio in AMED, and total fruits, total protein foods and fatty acid in HEI-2015 were linked to a decreased CD risk. Both diets were also associated with lower plasma inflammation biomarkers. Mediation analysis indicated that 7.66% and 13.40% of the reductions in CD risk attributed to AMED and HEI-2015 diets, respectively, were mediated by low-grade inflammation scores.

**Conclusions:**

Higher adherence to AMED and HEI-2015 might significantly reduce CD risk, partly due to their anti-inflammatory properties.

**Supplementary Information:**

The online version contains supplementary material available at 10.1186/s12916-024-03809-x.

## Background

Inflammatory bowel disease (IBD), including Crohn’s disease (CD) and ulcerative colitis (UC), is a chronic and relapsing inflammatory disorder affecting the gastrointestinal tract globally. The reported prevalence of IBD in 2019 has reached 4.9 million cases, with a rapid increase across various age and gender groups in both developed and developing countries, notably in Europe and North America [1, 2]. The etiology of IBD is complex and multifactorial, with genetic and environmental factors playing significant roles. Diet, among these factors, has been highlighted as one of the most influential environmental contributors, affecting the gut microbiota and influencing systemic inflammation and immune responses [3–5], which are associated with IBD development. However, these associations are observed after diagnosis, and no study has established direct causality.

Recent studies have suggested the potential benefits of specific nutrients, such as omega-3 fatty acids [6], dietary fiber [7, 8], and food items like fruits and vegetables [8] in mitigating the onset and symptoms of IBD. Meanwhile, several studies have indicated that the consumption of ultra-processed foods [9], high red meat consumption [10], and a pro-inflammation dietary pattern [11] were associated with an increased risk of CD or UC. However, due to the complex interplay of nutrients and foods, precise dietary recommendations for IBD prevention remain challenging. Notably, dietary guidelines, including the European Food-Based Dietary Guidelines (FBDGs) [12] and the Dietary Guidelines for Americans (DGAs) [13, 14], have shifted their focus from individual nutrients to healthy eating patterns for disease prevention, with various recommendations being made up to 2020 and updated in subsequent editions up to 2020–2025 [15, 16]. This dietary pattern approach better reflects real-world dietary practices and considers the cumulative effects of different dietary components and has been extensively studied in relation to chronic inflammatory diseases, such as rheumatoid arthritis [17], chronic obstructive pulmonary disease [18], cardiovascular diseases [19], and diabetes [20]. However, comprehensive research on the long-term effects of specific healthy eating patterns on IBD risk is still lacking.

To address this gap, we conducted a prospective analysis using data from the UK Biobank database. We selected four established healthy eating patterns, quantified by various dietary scores including the Alternate Mediterranean Diet (AMED) score, Healthy Eating Index 2015 (HEI-2015), Healthful Plant-based Diet Index (HPDI), and EAT-Lancet score, to analyze their associations with the risk of developing CD and UC. These dietary scores were selected because they represent comprehensive measures of adherence to healthy eating patterns, encompassing a wide range of dietary components known to impact inflammation and immune responses [21–23], both of which are associated with IBD development. These indices have also been extensively validated and widely used in epidemiological researches [19, 24–29], to assess the overall quality of diet but have distinct characteristics that capture different aspects of dietary patterns. For example, the AMED score is based on the Mediterranean diet, emphasizing high consumption of fruits, vegetables, whole grains, nuts, legumes, and olive oil, along with moderate intake of fish and low intake of red and processed meats, with light alcohol consumption [30]. The HEI-2015 assesses adherence to the Dietary Guidelines for Americans, promoting a diet rich in fruits, vegetables, whole grains, and lean proteins while limiting saturated fats, added sugars, and sodium [31] The HPDI emphasizes the health benefits associated with plant-based foods while minimizing consumption of animal-based products [8]. The EAT-Lancet score reflects a holistic approach to diet, considering both human health and environmental impact [32].

Low-grade inflammatory response refers to a subtle increase in peripheral pro-inflammatory markers, such as C-reactive protein (CRP), interleukin-6 (IL-6), and tumor necrosis factor-alpha (TNF-α), even in the absence of noticeable clinical symptoms. These markers have been implicated in the pathogenesis and disease activity of IBD [33–35]. Moreover, elevated levels of these markers have been associated with poorer treatment outcomes and prognosis in IBD patients. For example, a high baseline level of high-sensitivity CRP has been linked to a reduced remission rate in ulcerative colitis (UC) patients [36]. However, while inflammatory markers and scores are known to be associated with various diseases, their specific relationship with the risk of developing IBD remains unclear and warrants further investigation. It is also important to explore whether these markers mediate the association between diet and IBD risk, particularly considering the potential anti-inflammatory effects of certain dietary patterns, such as the Mediterranean diet [5]. Previous studies have suggested significant relationships between dietary patterns and changes in inflammatory biomarkers. However, these associations have primarily been identified in retrospective studies, with a notable lack of prospective investigations [4].

In the present study, we aimed to assess which dietary pattern demonstrates the most significant association with lower IBD risk and investigate potential relationships with anti-inflammatory mechanisms. By examining these associations, our study may provide valuable insights into optimal dietary recommendations for individuals at risk of IBD, contribute to the development of targeted dietary interventions for IBD prevention, and provide initial clues for further research on related mechanisms.

## Methods

### Study population

This study utilized data from the UK Biobank (https://www.ukbiobank.ac.uk) with application number 51671 [37], a comprehensive resource comprising detailed genetic and health information obtained from 500,000 individuals aged 40–69 years since 2006. The UK Biobank, established with the support of the UK Biobank Board and approved by the National Research Ethics Committee (REC ID: 16/NW/0274), serves as a valuable platform for investigating the interplay between genetic, environmental, and lifestyle factors in disease development. Participants in the UK Biobank provide extensive data, including demographic information, physical measurements, and biological samples (such as blood, urine, and saliva), along with detailed health and lifestyle questionnaires [38]. This rich dataset enables researchers to explore the intricate relationships between genetic and environmental factors in disease development [39] and to discover new biomarkers for early disease detection [40]. Similar studies utilizing UK Biobank data have been published, contributing to a growing body of literature examining various aspects of health and disease. Access to the UK Biobank data is granted to approved researchers upon payment and submission of study proposals for review and approval through the UK Biobank Access Management System. This ensures that research conducted using UK Biobank data is transparent, rigorous, and aligned with the principles of data protection and privacy. Detailed information about the UK Biobank can be found at https://www.ukbiobank.ac.uk/. Participants provided informed consent by signing consent forms. Our study analyzed a sample of 197,391 participants who completed at least one dietary questionnaire, excluding those with lost follow-up or self-reported/diagnosed IBD or cancer at baseline (Additional file 1: Fig. S1). We also excluded participants with implausible energy intake (< 500 kcal or > 5000 kcal), as previously suggested [21].

### Assessment of dietary scores

Dietary information was collected through the Oxford WebQ (www.ceu.ox.ac.uk/research/oxford-webq), a web-based 24-h recall questionnaire designed for large population studies. The validation of the Oxford WebQ has been previously confirmed [41]. Participants within the UK Biobank completed the Oxford WebQ on five separate occasions over 5 years, and mean values were computed from available data based on previous studies [42]. From April 2009 to September 2010, participants were invited to complete an online 24-h recall dietary questionnaire. A total of 70,655 individuals completed the first questionnaire. Subsequent follow-ups were conducted, with the first follow-up from February 2011 to April 2011 collecting 100,517 questionnaires, the second follow-up collecting 83,200 questionnaires (June 2011 to September 2011), the third follow-up collecting 103,698 questionnaires (October 2011 to December 2011), and the fourth follow-up collecting 100,168 questionnaires (April 2012 to June 2012). In this study, participants who completed at least one questionnaire, totaling 291,579 individuals, were included in the analysis. Participant responses for each dietary item were based on assigned portion sizes, with options like “None,” “1/2,” “1,” “2,” “3,” “4,” and “5 + ,” varying across items. Certain components like sodium, saturated fatty acids (SFAs), monounsaturated fatty acids (MUFAs), and polyunsaturated fatty acids (PUFAs) were indirectly estimated from food consumption [43]. During the calculation of dietary scores, original food portion sizes were also converted to grams using the same portion size definition as specified in the Oxford WebQ [44].

Detailed components and criteria for scoring each dietary pattern are available in Additional file 1: Tables S1–4, based on official definitions or prior studies [29, 30, 45, 46]. Specifically, the AMED score encompasses 9 components with a range of 0 to 9 [30]. HEI-2015 calculations involved converting food units to the appropriate units utilizing the Food Patterns Equivalents Database from US Department of Agriculture [47]. HEI-2015 is composed of 13 components, ranging from 0 to 100 [31]. The HPDI consists of 18 components within a range of 18 to 90 [48], and the EAT-Lancet score incorporates 14 components ranging from 0 to 14 [29]. Higher scores mean greater adherence to healthy eating patterns.

### Assessment of outcome

Participants were monitored by linking their data to the Health and Social Care Information Centre (in England and Wales) and the National Health Service Central Register (in Scotland). The study outcomes were the incidences of CD and UC, classified based on the International Classification of Diseases (ICD)−10 codes (K50 for CD, K51 for UC), identified through linkage to hospital inpatient database, primary care database, and cancer and death registries. This method of case ascertainment, using national health databases, provides a robust and validated approach, as confirmed by previous related study [49].

### Assessment of low-grade inflammation level

To evaluate the degree of low-grade inflammation, we introduced an aggregated index known as the low-grade inflammation score (INFLA-score), which has been validated in various studies [50, 51]. The INFLA-score is a novel scoring system that takes into account various biomarkers of systemic inflammation to quantify the concept of low-grade inflammation. This score integrates four distinct plasma inflammation biomarkers: white blood cell (WBC) count, platelet (PLT) count, C-reactive protein (CRP) level, and neutrophil-to-lymphocyte ratio (NLR) [52]. All biomarkers used to calculate the low-grade inflammation score were assessed at the time of participant recruitment, during the period from 2006 to 2010. These biomarkers were chosen based on their established roles in reflecting systemic inflammatory status and their relevance in previous research on inflammation and chronic diseases [53, 54]. Within this framework, each inflammation biomarker was allocated a score based on its position within the distribution. Specifically, scores ranging from 1 to 4 were assigned to biomarkers within the 7th to 10th deciles, while scores ranging from − 4 to 1 were assigned to those in the 1st to 4th deciles. The calculation of the INFLA-score involved assigning each of the four components a value from − 4 to 4 based on their respective deciles, with the summation of these values yielding an overall INFLA-score that could range from − 16 to 16. A higher INFLA-score represented an increased intensity of low-grade inflammation.

### Assessment of covariates

Participants provided self-reported information on sociodemographic factors (e.g., age, sex, ethnicity), lifestyle factors (e.g., smoking status, alcohol consumption, and daily sleeping time), medication intake (multivitamins, mineral supplements, aspirin, non-aspirin non-steroidal anti-inflammatory drug (NSAIDs), statins), and comorbidities (e.g., hypertension, hypercholesterolemia, and diabetes). Information on socioeconomic status, measured by index of multiple deprivation (IMD), and self-reported longstanding illness were also collected through the baseline questionnaire. Trained research staff measured participants’ height and weight to calculate their body mass index (BMI). Physical activity levels were assessed using the International Physical Activity Questionnaire-Short Form (IPAQ-SF) [55]. These covariates were selected as confounders in our analysis based on existing literature reporting their association with both the exposure and the risk of CD and UC [56, 57]. This method ensured that critical variables were considered in our analyses to mitigate potential confounding effects.

### Statistical analysis

In descriptive analyses, values were presented as either a mean (standard deviation) or number (percentage). Some variables, including the IMD, BMI, and physical activity, had missing values. To address this, we employed multiple imputation techniques to fill in the missing data. Our origin time is defined as the participant’s enrollment date in the UK Biobank, which occurred between 2006 and 2010. The start time in our survival analysis is the date when each participant first completed their dietary assessment. The end time corresponds to the first occurrence of events, including the diagnosis of CD or UC, death, or the end of follow-up (December 31, 2021). Person-years were computed from the date of the first dietary information assessment to CD/UC diagnosis, death, or the end of follow-up (December 31, 2021), whichever came first. We employed multivariable Cox regression models, taking age as the underlying timescale, to estimate hazard ratios (HRs) and 95% confidence intervals (CIs) for the association of healthy eating patterns with subsequent risk of incidence of CD and UC. The assumption of proportional hazards was tested by Schoenfeld tests in the UK Biobank and no violation of this assumption was found.

We stratified the analyses jointly by age, sex, and UK Biobank assessment centers in the basic model (model 1). To control for potential confounding by sociodemographic factors, lifestyle variables, medications, and health status, we additionally adjusted for ethnicity (white or other), index of multiple deprivation (continuous), BMI (continuous), smoking status (never, previous, current), alcohol consumption (special occasions only/never, 1–3 times a month, 1–4 times a week, daily or almost daily), physical activity (expressed in MET hours/week as a continuous variable), and daily sleep duration (continuous), as well as binary variables including medication use (multivitamins, mineral supplements, aspirin, non-aspirin NSAIDs, statins), comorbidities (e.g., hypertension, hypercholesterolemia, diabetes), and longstanding illness in model 2. To mitigate the possibility of reverse causality, participants who developed IBD in the initial years of follow-up were excluded, as we previously reported [49]. We utilized restricted cubic splines with four knots at the 5th, 35th, 65th, and 95th centiles to model the potential non-linear associations between dietary scores and the risk of CD and UC. This choice aligns with recommendations from Harrell’s “Regression Modeling Strategies” [58], which suggests that four knots provide a balance between model fit and smoothness while avoiding overfitting. Cubic splines are a flexible and powerful tool for capturing complex, nonlinear associations in data by fitting a series of cubic polynomials between predefined knots [59]. By utilizing cubic splines, we were able to accurately assess the shape of the relationship between each estimated dietary score and the outcome variables. This method allows us to explore potential non-linear relationships between dietary patterns and the risk of IBD, providing a more nuanced understanding of how different levels of dietary adherence may impact IBD risk.

We conducted several sensitivity analyses to test the robustness of our findings. Firstly, we excluded IBD cases that occurred within the first 2 years of follow-up to mitigate any potential reverse causation effects. Secondly, given that Oxford WebQ relies on diet recall for a single day and may not fully represent participants’ typical dietary habits [42], we performed a sensitivity analysis that excluded participants reporting atypical diets during any of the five follow-up assessments. Thirdly, considering the association between dietary intake and BMI, which can influence health outcomes [60], we examined the potential over-adjustment bias by comparing associations estimated from models both with and without BMI categories. Fourthly, digestive system disorders may affect the dietary habits of participants. Therefore, we are excluding participants with pre-existing digestive system disorders at baseline and those who developed digestive system disorders during the period between baseline and completion of the dietary questionnaire. Lastly, as covariates were collected prior to dietary assessments, there is a potential for temporal mismatch that might introduce variability in the results. To minimize this risk, we conducted sensitivity analyses excluding participants with a time gap of more than 2 years between covariate and dietary data collection. Furthermore, to explore potential effect modification, we analyzed whether the association between healthy diet patterns and IBD risk varied across subgroups defined by sex, age, obesity, smoking, drinking, physical activity, and regular NSAIDs use. Interaction tests were conducted by introducing dietary scores and these covariates as multiplicative interaction terms in our models. We did not perform multiple testing correction in the present study.

Mediation analysis allows for the decomposition of the total effect of an exposure on an outcome into direct and indirect effects, with the indirect effect operating through one or more mediator variables [61]. This approach enables the estimation of the natural direct and indirect effects, as well as the proportion mediated, while accounting for potential confounding factors. By conducting mediation analyses, it provides insight into how much of the association between diet and IBD risk can be explained by changes in inflammatory biomarkers, thus highlighting the importance of inflammation as a potential mediator. To investigate whether inflammation mediated the association of healthy eating patterns with the risk of CD and UC, we first conducted the linear regression analysis to confirm the association of healthy eating patterns with INFLA-score and four inflammation biomarkers. Subsequently, we conducted multivariable Cox regression analysis to verify the correlation between the INFLA-score, the four inflammation biomarkers, and the onset of CD and UC. Next, we conducted multivariable Cox regression analysis to confirm the association of INFLA-score and four inflammation biomarkers with the incidences of CD and UC. Finally, we estimated the proportion of the total association mediated through inflammation using the “mediator” package in R. Statistical analysis was conducted under the guidance and supervision of experienced biostatisticians with expertise in epidemiological research methods and statistical techniques. We performed all analyses using the R software (version 3.5.0, R Foundation for Statistical Computing, Vienna, Austria).

## Results

### Study population

A total of 197,391 participants from the UK Biobank were included in this study, from 2006 to 2010 (Additional file 1: Fig. S1). Table 1 presents the characteristics of the study participants based on the distribution of dietary scores. Participants with higher dietary scores tended to be female, engaged in regular exercise, had a lower BMI and IMD, and tended not to have hypercholesterolemia, diabetes, or longstanding illness. Additionally, those with higher scores were more inclined to use vitamin or mineral supplements and less likely to use medications such as aspirin, NSAIDs, or statins.

**Table 1:** Baseline characteristics across healthy eating patterns quartiles among UK Biobank participants

Characteristics	AMED score			HEI-2015 tertile			HPDI tertile			EAT-Lancet tertile		
	**0–3**	**4–5**	**6–9**	**Q1**	**Q2**	**Q3**	**Q1**	**Q2**	**Q3**	**Q1**	**Q2**	**Q3**
No. of participants	92,598	70,255	34,538	65,797	65,797	65,797	59,051	66,239	72,101	55,905	55,675	85,811
Mean (SD)^a^ age, years	56.1 (8.07)	56.6 (7.87)	56.8 (7.76)	55.5 (8.16)	56.5 (7.95)	57.2 (7.64)	55.6 (8.09)	56.5 (7.95)	56.9 (7.78)	56.7 (7.90)	56.5 (7.97)	56.2 (7.96)
Female, *N* (%)	48,632 (52.5)	38,767 (55.2)	20,525 (59.4)	31,666 (48.1)	35,426 (53.8)	40,832 (62.1)	27,772 (47.0)	35,666 (53.8)	44,486 (61.7)	26,783 (47.9)	29,899 (53.7)	51,242 (59.7)
White, *N* (%)	87,962 (95.0)	67,398 (95.9)	33,443 (96.8)	62,448 (94.9)	62,968 (95.7)	63,387 (96.3)	56,680 (96.0)	63,503 (95.9)	68,620 (95.2)	54,213 (97.0)	53,464 (96.0)	81,126 (94.5)
Mean (SD) index of multiple deprivation	16.2 (12.9)	15.0 (12.0)	14.3 (11.4)	16.3 (13.0)	15.2 (12.2)	14.7 (11.7)	15.7 (12.6)	15.4 (12.4)	15.2 (12.1)	15.5 (12.5)	15.4 (12.3)	15.4 (12.2)
Mean (SD) BMI, kg/m^2^	27.4 (4.74)	26.8 (4.58)	26.2 (4.44)	27.2 (4.70)	27.0 (4.67)	26.6 (4.57)	27.6 (4.90)	27.0 (4.59)	26.4 (4.43)	27.4 (4.75)	27.1 (4.65)	26.6 (4.56)
Never smoker, *N* (%)	50,815 (54.9)	40,597 (57.8)	20,975 (60.7)	36,354 (55.3)	37,621 (57.2)	38,412 (58.4)	33,994 (57.6)	37,580 (56.7)	40,813 (56.6)	30,882 (55.2)	31,603 (56.8)	49,902 (58.2)
Never drinker, *N* (%)	16,052 (17.3)	11,231 (16.0)	4576 (13.2)	10,839 (16.5)	10,463 (15.9)	10,557 (16.0)	9510 (16.1)	10,305 (15.6)	12,044 (16.7)	8147 (14.6)	8749 (15.7)	14,963 (17.4)
Median (IQR)^b^ physical activity, MET hours/week	41.6 (42.5)	41.3 (40.2)	42.5 (39.6)	41.5 (42.8)	41.5 (40.7)	42.0 (39.9)	39.6 (40.5)	41.4 (41.1)	43.7 (41.7)	40.2 (40.6)	41.4 (41.3)	42.8 (41.4)
Above 8 h of daily sleeping time, *N* (%)	69,960 (75.6)	54,589 (77.7)	27,296 (79.0)	49,643 (75.4)	50,692 (77.0)	51,510 (78.3)	45,279 (76.7)	51,026 (77.0)	55,540 (77.0)	43,112 (77.1)	43,001 (77.2)	65,732 (76.6)
Hypertension, *N* (%)	52,082 (56.2)	38,839 (55.3)	18,330 (53.1)	36,133 (54.9)	36,702 (55.8)	36,416 (55.3)	33,838 (57.3)	36,816 (55.6)	38,597 (53.5)	32,845 (58.8)	31,003 (55.7)	45,403 (52.9)
Hypercholesterolemia, *N* (%)	15,961 (17.2)	11,493 (16.4)	4997 (14.5)	10,903 (16.6)	10,923 (16.6)	10,625 (16.1)	10,075 (17.1)	10,887 (16.4)	11,489 (15.9)	10,142 (18.1)	9295 (16.7)	13,014 (15.2)
Diabetes, *N* (%)	4986 (5.4)	3103 (4.4)	1217 (3.5)	3334 (5.1)	3096 (4.7)	2876 (4.4)	2937 (5.0)	3107 (4.7)	3262 (4.5)	2883 (5.2)	2695 (4.8)	3728 (4.3)
Longstanding illness, *N* (%)	26,668 (28.8)	19,478 (27.7)	9053 (26.2)	18,919 (28.8)	18,324 (27.8)	17,956 (27.3)	17,438 (29.5)	18,359 (27.7)	19,402 (26.9)	16,405 (29.3)	15,529 (27.9)	23,265 (27.1)
Multivitamin use, *N* (%)	13,154 (14.2)	10,964 (15.6)	5794 (16.8)	8897 (13.5)	9864 (15.0)	11,151 (16.9)	8150 (13.8)	9814 (14.8)	11,948 (16.6)	7739 (13.8)	8336 (15.0)	13,837 (16.1)
Intake of mineral supplements, *N* (%)	19,968 (21.6)	16,267 (23.2)	8316 (24.1)	13,868 (21.1)	14,692 (22.3)	15,991 (24.3)	12,517 (21.2)	14,639 (22.1)	17,395 (24.1)	11,608 (20.8)	12,160 (21.8)	20,783 (24.2)
Aspirin use, *N* (%)	11,725 (12.7)	8735 (12.4)	3994 (11.6)	8198 (12.5)	8210 (12.5)	8046 (12.2)	7605 (12.9)	8171 (12.3)	8678 (12.0)	7649 (13.7)	6931 (12.4)	9874 (11.5)
Non-aspirin NSAIDs use, *N* (%)	16,256 (17.6)	11,370 (16.2)	5258 (15.2)	11,638 (17.7)	10,996 (16.7)	10,250 (15.6)	10,237 (17.3)	11,107 (16.8)	11,540 (16.0)	9109 (16.3)	9298 (16.7)	14,477 (16.9)
Statin use, *N* (%)	13,858 (15.0)	9941 (14.1)	4303 (12.5)	9492 (14.4)	9466 (14.4)	9144 (13.9)	8810 (14.9)	9477 (14.3)	9815 (13.6)	8929 (16.0)	8047 (14.5)	11,126 (13.0)

Abbreviations: *SD *Standard deviation, *IQR *Interquartile range, *Q *Quantile, *BMI *Body mass index, *MET hours/week *Hours of physical activity per week, *NSAIDs *Non-steroidal anti-inflammatory drugs, *AMED *Alternate Mediterranean Diet, *HEI-2015 *Healthy Eating Index 2015, *HPDI *Healthful Plant-based Diet Index

^a^We use the means (SD) for normally distributed variables and the medians (IQR) for non-normally distributed variables

^b^Interquartile range (IQR): the range from values of the 25th to the 75th deciles

### Healthy dietary patterns and risk of IBD

**Fig. 1:**
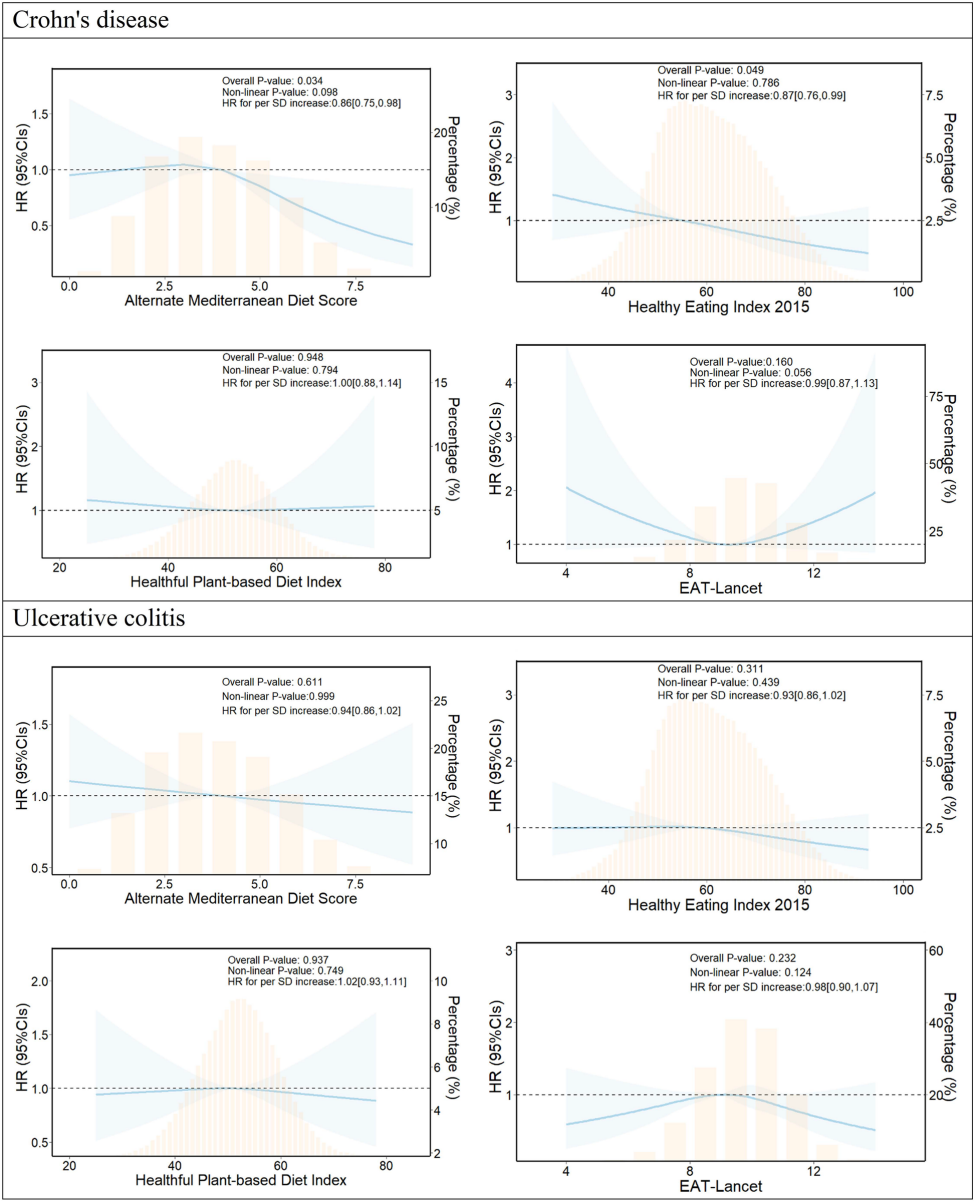
Estimated nonlinear association between healthy eating patterns and risk of Crohn’s disease and ulcerative colitis. Abbreviation: HR, hazard ratio; CI, confidence interval; SD, standard deviation. Separate models were fitted for Crohn’s disease and ulcerative colitis with a restricted cubic spline for each healthy eating pattern. All models were adjusted for sociodemographic characteristics, lifestyle factors, medications, and comorbidities (see footnote in Table 2). Shaded areas represent 95% confidence intervals

During up to the 2,193,436 person-years of follow-up, 260 CD cases and 601 UC cases were documented (Table 2). The results revealed that higher scores on the AMED and the HEI-2015 were associated with a reduced risk of CD (*P* trend < 0.05). Specifically, compared to participants in the lowest category of AMED score, those with AMED scores of 4–5 and 6–9 had age and gender-adjusted HRs of 0.87 (95% CI, 0.66–1.14) and 0.51 (95% CI, 0.33–0.78), respectively, for CD. These associations remained consistent even after adjusting for potential confounders, including sociodemographic factors, lifestyle factors, medication intake, and comorbidities. Similar trends were observed for the association of HEI-2015 category with CD risk. For each SD increase in AMED and HEI-2015 scores, the risk of CD decreased by 0.86 (95% CI, 0.75–0.98) and 0.87 (95% CI, 0.76–0.99), respectively, with no evidence against nonlinearity (Fig. 1). There was insufficient evidence to support an association between the HPDI score and the EAT-Lancet score with CD risk. Furthermore, no sufficient evidence of association was found between any of the four healthy eating patterns and the risk of UC.

**Table 2:** Association between healthy eating patterns and risk of Crohn’s disease and ulcerative colitis

Variables	Cases	Person-years	Incidence rate^a^	Age and gender-stratified HR [95% CI]^b^	Multivariable-adjusted HR [95% CI]^c^
**Crohn’s disease**					
AMED score					
0–3	141	1,025,234	13.8	1.00 [reference]	1.00 [reference]
4–5	89	782,806	11.4	0.87 [0.66, 1.14]	0.92 [0.70, 1.21]
6–9	30	385,396	7.8	0.51 [0.33, 0.78]	0.49 [0.31, 0.77]
* P* trend				0.014	0.024
Per SD increase				0.85 [0.75, 0.97]	0.86 [0.75, 0.98]
HEI-2015					
Q1	108	727,931	14.8	1.00 [reference]	1.00 [reference]
Q2	83	731,752	11.3	0.81 [0.60, 1.09]	0.82 [0.61, 1.10]
Q3	69	733,753	9.4	0.65 [0.47, 0.89]	0.65 [0.47, 0.90]
* P* trend				0.029	0.032
Per SD increase				0.87 [0.76, 0.99]	0.87 [0.76, 0.99]
HPDI					
Q1	73	656,081	11.1	1.00 [reference]	1.00 [reference]
Q2	83	735,836	11.3	1.04 [0.75, 1.44]	1.06 [0.76, 1.48]
Q3	104	801,519	13	1.20 [0.88, 1.65]	1.24 [0.90, 1.72]
* P* trend				0.877	0.958
Per SD increase				0.99 [0.87, 1.12]	1.00 [0.88, 1.14]
EAT-Lancet score					
Q1	73	621,822	11.7	1.00 [reference]	1.00 [reference]
Q2	66	618,605	10.7	0.88 [0.62, 1.25]	0.88 [0.62, 1.26]
Q3	121	953,008	12.7	1.08 [0.80, 1.45]	1.09 [0.80, 1.48]
* P* trend				0.779	0.909
Per SD increase				0.98 [0.86, 1.12]	0.99 [0.87, 1.13]
**Ulcerative colitis**					
AMED score					
0–3	307	1,024,461	30	1.00 [reference]	1.00 [reference]
4–5	200	782,244	25.6	0.85 [0.70, 1.02]	0.90 [0.75, 1.09]
6–9	94	385,115	24.4	0.76 [0.60, 0.98]	0.82 [0.64, 1.07]
* P* trend				0.020	0.155
Per SD increase				0.90 [0.83, 0.98]	0.94 [0.86, 1.02]
HEI-2015					
Q1	213	727,460	29.3	1.00 [reference]	1.00 [reference]
Q2	211	731,111	28.9	0.96 [0.79, 1.18]	1.01 [0.82, 1.24]
Q3	177	733,248	24.1	0.80 [0.65, 0.99]	0.86 [0.69, 1.07]
* P* trend				0.020	0.124
Per SD increase				0.90 [0.83, 0.98]	0.93 [0.86, 1.02]
HPDI					
Q1	181	655,559	27.6	1.00 [reference]	1.00 [reference]
Q2	208	735,268	28.3	1.11 [0.90, 1.37]	1.13 [0.91, 1.40]
Q3	212	800,993	26.5	1.03 [0.83, 1.28]	1.08 [0.87, 1.34]
* P* trend				0.993	0.714
Per SD increase				1.00 [0.92, 1.09]	1.02 [0.93, 1.11]
EAT-Lancet score					
Q1	164	621,365	26.4	1.00 [reference]	1.00 [reference]
Q2	191	618,087	30.9	1.20 [0.96, 1.49]	1.21 [0.97, 1.51]
Q3	246	952,367	25.8	0.98 [0.79, 1.21]	1.02 [0.83, 1.27]
* P* trend				0.395	0.721
Per SD increase				0.96 [0.89, 1.05]	0.98 [0.90, 1.07]

Abbreviations: *HR *Hazard ratio, *CI *Confidence interval, *SD *Standard deviation, *Q *Quantile, *AMED *Alternate Mediterranean Diet, *HEI-2015 *Healthy Eating Index 2015, *HPDI *Healthful Plant-based Diet Index

^a^Per 100,000 person-years

^b^Hazard ratio and 95% CI were estimated from crude Cox regression model stratified by sex, age, and assessment center

^c^Fully adjusted model additionally adjusted for sociodemographic characteristics (ethnicity, index of multiple deprivations, and BMI), lifestyle factor (smoking status, alcohol consumption, physical activity, and daily sleeping time), medications (multivitamins, mineral, aspirin, non-aspirin NSAIDs, and statins use), and comorbidities (hypercholesterolemia, hypertension, diabetes, and longstanding illness)

These primary results remained robust in sensitivity analyses (Additional file 1: Table S5). Lagging the exposure by 2 years or considering only participants who reported their typical diet did not substantially alter the associations between healthy eating pattern scores and the risk of CD and UC. The associations remained stable when not adjusting for BMI categories, and after excluding participants with pre-existing digestive system disorders at baseline and those who developed them between baseline and the completion of the dietary questionnaire. The sensitivity analyses, excluding participants with covariate-dietary assessment time gaps exceeding 2 years, yielded consistent results. We further explored potential effect modification across various subgroups defined by sex, age, obesity, smoking, drinking, physical activity, and regular NSAIDs use, and no significant evidence for effect modification was observed (all* P*-interaction > 0.05, Additional file 1: Tables S6–7).

**Fig. 2:**
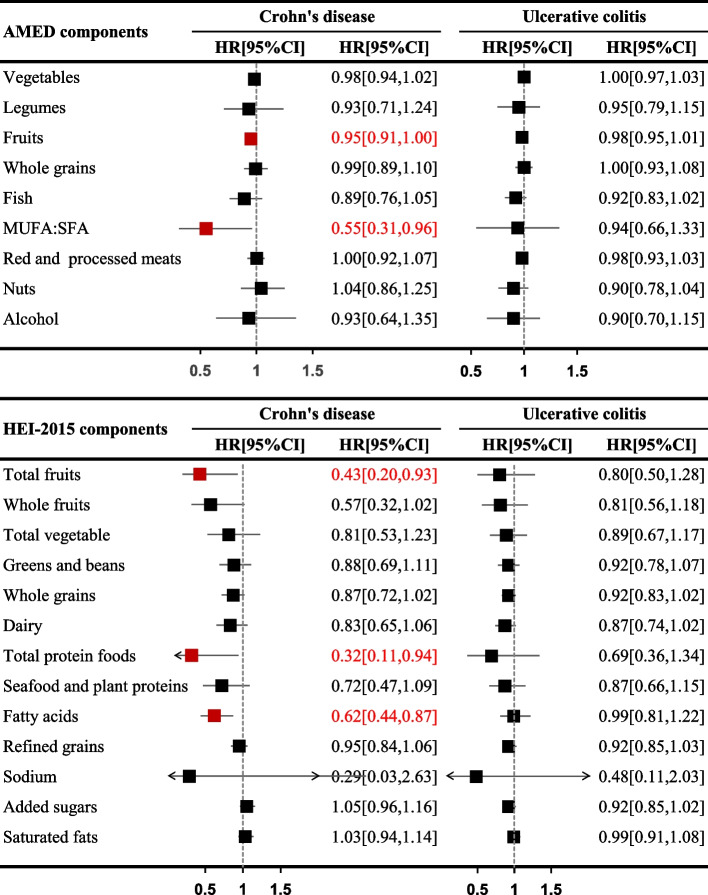
Association between each food group of AMED and HEI-2015 with risk of CD and UC. Abbreviation: CD, Crohn’s disease; UC, ulcerative colitis; HR, hazard ratio; CI, confidence interval; AMED, Alternate Mediterranean Diet; HEI-2015, Healthy Eating Index 2015; MUFA, monounsaturated fatty acid, SFA, saturated fatty acid. Estimated effects were based on the fully adjusted model (see footnote in Table 2)

Given the significant associations of AMED and HEI-2015 scores with the reduced risk of CD, we conducted further investigations into the individual components of these scores (Fig. 2). Per SD increase in the fruits and MUFA:SFA ratio within the AMED component were associated with 5% (HR = 0.95, 95% CI 0.91–1.00) and 45% (HR = 0.55, 95% CI 0.31–0.96) lower risk of CD, respectively. Meanwhile, in the HEI-2015 component, higher intake of total fruits (HR = 0.43, 95% CI 0.20–0.93), total protein foods (HR = 0.32, 95% CI 0.11–0.94), and fatty acids (defined as (MUFA + PUFA):SFA, HR = 0.62, 95% CI 0.44–0.87) were associated with a decreased risk of CD.

### Mediation analyses of inflammation in the association of healthy diet patterns with IBD risk

**Fig. 3:**
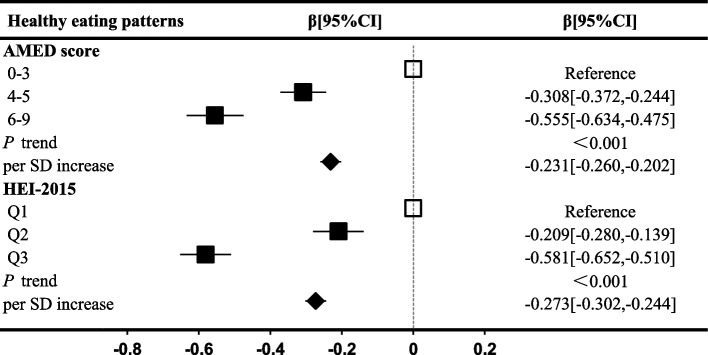
Association of healthy eating patterns with low-grade inflammation scores. Abbreviation: CI, confidence interval; SD, standard deviation; Q, quantile; AMED, Alternate Mediterranean Diet; HEI-2015, Healthy Eating Index 2015; HPDI, Healthful Plant-based Diet Index. Regression coefficient beta and 95% CI were estimated from multivariate-linear regression models stratified by sex, age, and assessment center and adjusted for sociodemographic characteristics (ethnicity, index of multiple deprivations, and BMI), lifestyle factor (smoking status, alcohol consumption, physical activity, and sleep quality), medication (multivitamins, mineral, aspirin, non-aspirin NSAIDs, and statins use), and baseline disease (hypercholesterolemia, hypertension, diabetes, and longstanding illness)

To explore potential mechanisms underlying the association between healthy dietary patterns and IBD risk, we estimated the association of AMED and HEI-2015 scores with markers of inflammation. In the fully adjusted model, higher healthy dietary scores were inversely associated with all plasma inflammatory factors and cumulative INFLA-score (Fig. 3 and Additional file 1: Table S8). For instance, participants with AMED scores of 4–5 and 6–9 had a decrease of − 0.308 (95% CI, − 0.372 to − 0.244) and − 0.555 (95% CI, − 0.634 to − 0.475), respectively, in the INFLA-score compared to those in the lowest category of AMED score. Continuous trends were also significant, with each SD increase in AMED and HEI-2015 associated with a decrease of − 0.231 (95% CI, − 0.260 to − 0.202) and − 0.273 (95% CI, − 0.302 to − 0.244) in the INFLA-score, respectively (Fig. 3).

Next, we examined the relationships between inflammatory factors and the long-term risk of CD and UC (Additional file 1: Table S9). A higher INFLA-score, along with elevated levels of WBC, CRP, and NLR, was found to be statistically significantly associated with an increased risk of CD and UC. No significant association was found between PLT and CD or UC risk.

**Fig. 4:**
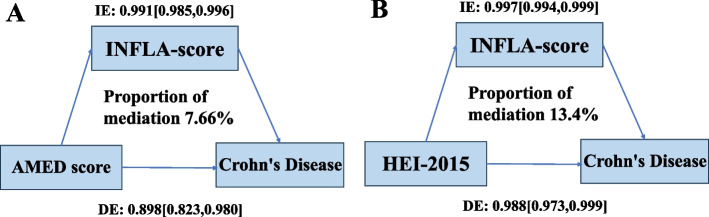
Mediation proportion of the association between healthy eating patterns and CD risk mediated by INFLA-score. Abbreviations: CD, Crohn’s disease; INFLA-score, low-grade inflammation score; AMED, Alternate Mediterranean Diet; HEI-2015, Healthy Eating Index 2015. All models were adjusted for sociodemographic characteristics, lifestyle factors, medications, and comorbidities (see footnote in Table 2)

Finally, mediation analyses were performed to explore the potential role of inflammation in mediating the associations between AMED and HEI-2015 diets and reduced CD risk. The results showed that low-grade inflammation partially mediated the associations. Specifically, 7.66% of the reduced CD risk for AMED diet and 13.40% for HEI-2015 diet were mediated through the INFLA-score (Fig. 4).

## Discussion

In this large prospective cohort of middle-aged men and women in UK, we comprehensively investigated the link between healthy dietary patterns and the risk of IBD. Our findings demonstrated that higher adherence to the AMED and HEI-2015 was associated with a reduced risk of CD but not UC. These findings were consistent across multiple sensitivity and subgroup analyses. Furthermore, we identified specific components of these dietary patterns, such as the fruits and MUFA:SFA ratio in AMED, and total fruits, total protein foods and fatty acids in HEI-2015, that were significantly associated with a decreased risk of CD. Notably, our analysis indicated a potential association between the anti-inflammatory properties of the AMED and HEI-2015 diets and reduced CD risk. These findings suggest that adopting healthy dietary patterns may hold promise in preventing CD, warranting further investigation and potential dietary interventions for IBD prevention.

Prior investigations into the association between healthy eating patterns and the risk of IBD have yielded inconsistent findings. For instance, a recent investigation based on two Swedish cohorts found no substantial association between HEI-2015 and the risk of older-onset CD. However, this study also reported that, compared to the lowest quartile, the modified Mediterranean Diet Score (mMED) with the highest quartile has an age-adjusted HR of 0.54 (95% CI, 0.30–0.96), while the HPDI with the highest quartile has a multivariable-adjusted HR of 0.52 (95% CI, 0.32–0.85) [62], which partially aligns with our findings. Similarly, within the Lifelines cohort, AMED scores and adherence to the HEI-2015 were negatively associated with CD risk, although it was not statistically significant [63]. Conversely, a case–control study reported a lower odds ratio of 0.34 for UC (95% CI, 0.12–0.96), but not CD, among individuals with higher HEI-2015 scores [64]. These inconsistencies among studies may be, at least in part, attributed to the diversity in study populations, study designs, sample sizes, and methods used to assess dietary patterns. It is worth noting that conclusive health effect associations for the EAT-Lancet diet have yet to be established based on prior research. Further research is needed to verify the relationship of the EAT-Lancet diet on IBD risk.

Among several healthy dietary patterns, AMED and HEI-2015 were associated with a reduced risk of CD, while other dietary patterns did not show this link. This difference may be attributed to the unique characteristics of AMED and HEI-2015, compared to other dietary patterns. Both the Mediterranean diet and HEI-2015 emphasize the intake of unsaturated fatty acids. Omega-3 PUFAs, a sort of unsaturated fatty acids, as an inhibitor of arachidonic acid metabolism in pharmacological mechanisms, the anti-inflammatory effect of n-3 PUFA has been confirmed in animal models to have certain therapeutic effects on inflammatory diseases such as rheumatoid arthritis [65] and IBD [66]. Our study also found that this component was related to a decreased risk of CD (Fig. 2). Additionally, the Mediterranean diet encourages moderate alcohol consumption and the use of olive oil, which contains a variety of polyphenolic substances and has potential anti-inflammatory properties [67, 68]. The exact mechanisms underlying the association between AMED and HEI-2015 with reduced CD risk require further research to elucidate.

Consistent with prior studies demonstrating a protective effect of dietary fiber and unsaturated fatty acid intake against IBD risk, our study has suggested a significant reduction in CD risk associated with specific components (i.e., fruits and MUFA:SFA ratio) of the AMED. These findings align with previous research [5, 8, 69, 70] and are further supported by our observation of a negative association between total fruit intake and the ratio of (PUFA + MUFA) to SFA within the HEI-2015. Fruits are known to be rich sources of dietary fiber, antioxidants, vitamins, and minerals, all of which possess potential anti-inflammatory properties [71]. A previous meta-analysis suggested an association between fruit consumption and a reduced risk of CD [8]. MUFAs prominently found in sources like olive oil, canola oil, and peanut oil [72] have been associated with a lowered CD risk in a previous cohort study [69]. The potential therapeutic benefits of olive oil for IBD patients have been documented in animal and in vitro studies [70]. The anti-inflammatory potential of PUFAs, particularly the well-recognized omega-3 PUFAs, has garnered significant attention due to their ability to inhibit arachidonic acid metabolism, an important pathway of inflammation [73]. Abundantly present in oily fish such as salmon, trout, and herring [72], these omega-3 PUFAs have been linked to a reduced CD risk [74]. Interestingly, our study also identified a protective component against CD in the total protein foods category, encompassing meat, poultry, eggs, seafood, nuts, and legumes as defined in HEI-2015. However, there is inconsistent with existing research on the relationship between this aggregated component and IBD [75–77]. Additionally, it does not differentiate between plant protein and animal protein, limiting our ability to determine which type of protein is associated with the observed outcomes. This disparity highlights the importance of prospective investigations in further confirming and enhancing the validity of our findings.

The justification for conducting the mediation analysis in our study is based on the causal hypothesis that dietary patterns may influence the risk of IBD through their effects on low-grade inflammation. Our finding of an association between the AMED score and low inflammation scores aligns with Bonaccio et al.’s study [5], confirming the external validity of our results and highlighting the anti-inflammatory benefits of the Mediterranean diet. Both AMED and HEI-2015 diets are rich sources of anti-inflammatory components, such as omega-3 fatty acids, fruits, vegetables, and whole grains, which have consistently been linked to a reduction in the occurrence of IBD and the alleviation of its symptoms [78]. Moreover, these health-promoting dietary regimens also restrict the consumption of pro-inflammatory items, including red and processed meat, sugar-sweetened beverages, and high-fat dairy products [79], resulting in a strong negative correlation with plasma inflammatory biomarkers as observed in our study (WBC, PLT, CRP, NLR, and the aggregated INFLA-score). This alignment with previous research reinforces the well-established association between plant-based dietary patterns and reduced inflammation [80]. To quantify the extent to which inflammation mediates the relationship between diet and IBD risk, we performed a mediation analysis. Our results indicated that the proportion of mediation by low-grade inflammation was 7.66% for the association between the AMED score and CD risk and 13.40% for the HEI-2015 score and CD risk. These values suggest that while low-grade inflammation plays a role in mediating the negative association of these dietary patterns, other mechanisms are also likely involved. For instance, AMED and HEI-2015 may exert influence on other mechanisms critical to the development of IBD, including the modulation of the gut microbiota and the maintenance of intestinal barrier integrity [81]. Thus, to attain a comprehensive understanding of these intricate mechanisms, further in-depth investigation is warranted.

The strengths of this study lie in its prospective design with a large sample size, high-quality data source, and robust adjustment for confounding factors, facilitating a direct comparison of multiple dietary patterns for IBD. We also explored associations with constituent components, and further provided insights into the potential mechanistic pathways through which these dietary patterns were linked to the protection against Crohn’s disease. However, certain limitations must be acknowledged. Firstly, our study population primarily consisted of middle-aged participants, which may raise concerns about the generalizability of our findings to younger individuals at risk of IBD. Nonetheless, our investigation remains valuable in identifying modifiable risk factors for elderly-onset disease, considering that environmental factors may play a more significant role in IBD [82]. Moreover, it is important to note that many of the beneficial effects of health diet patterns, such as the Mediterranean diet, have specifically been demonstrated in older adults [83]. Secondly, the use of a 24-h recall for dietary measurements may not fully capture participants’ typical intake and could be subject to recall bias [84]. The timing of when participants started to consume a particular diet is also unknown. However, prior validation studies have demonstrated that dietary data collected using the Oxford WebQ provide reasonably valid estimates of dietary intake [41]. Furthermore, we conducted sensitivity analyses considering only participants who reported their typical diet in the questionnaire, and the results remained consistent with the original findings. Thirdly, the lack of updated dietary data during the follow-up period is a limitation. However, evidence from other studies suggests that individuals’ dietary intake tends to remain relatively stable over time [85], reducing the likelihood of significant changes in their diet categorization. Fourthly, the “healthy volunteer” effect in the UK Biobank may limit the generalizability of our findings, but the large size and heterogeneity of exposure measures in the UK Biobank allow for valid scientific inferences of exposure-outcome relationships that are applicable to other populations [86]. Fifthly, despite our comprehensive adjustment for confounders, residual confounding effects cannot be entirely ruled out. Lastly, as with any observational study, causation cannot be established. Further research, including interventional studies, is required to confirm the causal relationship between these dietary patterns and IBD risk.

## Conclusions

In conclusion, our study suggests a possible link between adherence to healthy dietary patterns, such as the AMED and the HEI-2015, and a reduced risk of CD. It is important to note that our study is observational in nature, and causation cannot be inferred. Our study also highlights the potential role of inflammation as a mediator in the relationship between healthy dietary patterns and CD risk. This potentially underscores the feasibility and mechanisms of dietary interventions in IBD prevention. Our findings may have implications for public health initiatives aimed at promoting healthy eating habits, but further research, including interventional studies, is needed to establish causal relationships and elucidate the underlying mechanisms.

## Supplementary Information

Supplementary Material 1.

## Data Availability

This research got access to the UK Biobank (https://www.ukbiobank.ac.uk) under application number 51671. Further information is available from the corresponding author upon request.

## Abbreviations

AMED: Alternate Mediterranean Diet
BMI: Body mass index
CD: Crohn’s disease
CI: Confidence interval
CRP: C-reactive protein
DGAs: Dietary Guidelines for Americans
FBDGs: European Food-Based Dietary Guidelines
HEI-2015: Healthy Eating Index 2015
HPDI: Healthful Plant-based Diet Index
HR: Hazard ratio
IBD: Inflammatory bowel disease
ICD: International Classification of Diseases
IL-6: Interleukin-6
IMD: Index of multiple deprivation
INFLA-score: Low-grade inflammation score
IPAQ-SF: International Physical Activity Questionnaire-Short Form
MD: Mediterranean diet
mMED: Modified Mediterranean Diet Score
MUFAs: Monounsaturated fatty acids
NLR: Neutrophil-to-lymphocyte ratio
NSAIDs: Non-steroidal anti-inflammatory drugs
PLT: Platelet
PUFAs: Polyunsaturated fatty acids
SD: Standard deviation
SFAs: Saturated fatty acids
TNF-α: Tumor necrosis factor-alpha
UC: Ulcerative colitis
WBC: White blood cell

## Availability of data and materials

The data that support the findings of this study are available from UK Biobank project site, subject to registration and application process. Further details can be found at https://www.ukbiobank.ac.uk. The code used in this study is available upon reasonable request to the corresponding author.
