# Supplementary material for: Healthy eating patterns associated with reduced risk of inflammatory bowel disease by lowering low-grade inflammation: evidence from a large prospective cohort study
Source: BMC Med. 2024 Dec 18;22:589. doi: 10.1186/s12916-024-03809-x (PMC11658349; doi:10.1186/s12916-024-03809-x)
Supplement: Supplementary file 1 — Supplementary Material 1. [file 12916_2024_3809_MOESM1_ESM.docx]

**Supplemental Materials**

| **Contents** | **Page** |
| --- | --- |
| **Fig. S1** Flow diagram of participants in the present study. | 2 |
| **Table S1** Components and scoring criteria of the alternate Mediterranean Diet (AMED) Score. | 3 |
| **Table S2** Components and scoring criteria of the Healthy Eating Index 2015 (HEI-2015). | 4-5 |
| **Table S3** Components and scoring criteria of the Healthful Plant-based Diet Index (HPDI). | 6-7 |
| **Table S4** Components and scoring criteria of EAT-Lancet Score. | 8 |
| **Table S5** Sensitivity analyses of association between healthy eating score and risk of Crohn’s disease and ulcerative colitis. | 9-10 |
| **Table S6** Subgroup analyses of association between per SD increase of healthy eating score and risk of Crohn’s disease. | 11 |
| **Table S7** Subgroup analyses of association between per SD increase of healthy eating score and risk of Ulcerative colitis. | 12 |
| **Table S8** Associations of per SD score of healthy eating patterns with inflammation biomarkers. | 13 |
| **Table S9** Association between inflammatory factors and the risk of Crohn’s disease and ulcerative colitis. | 14 |

**
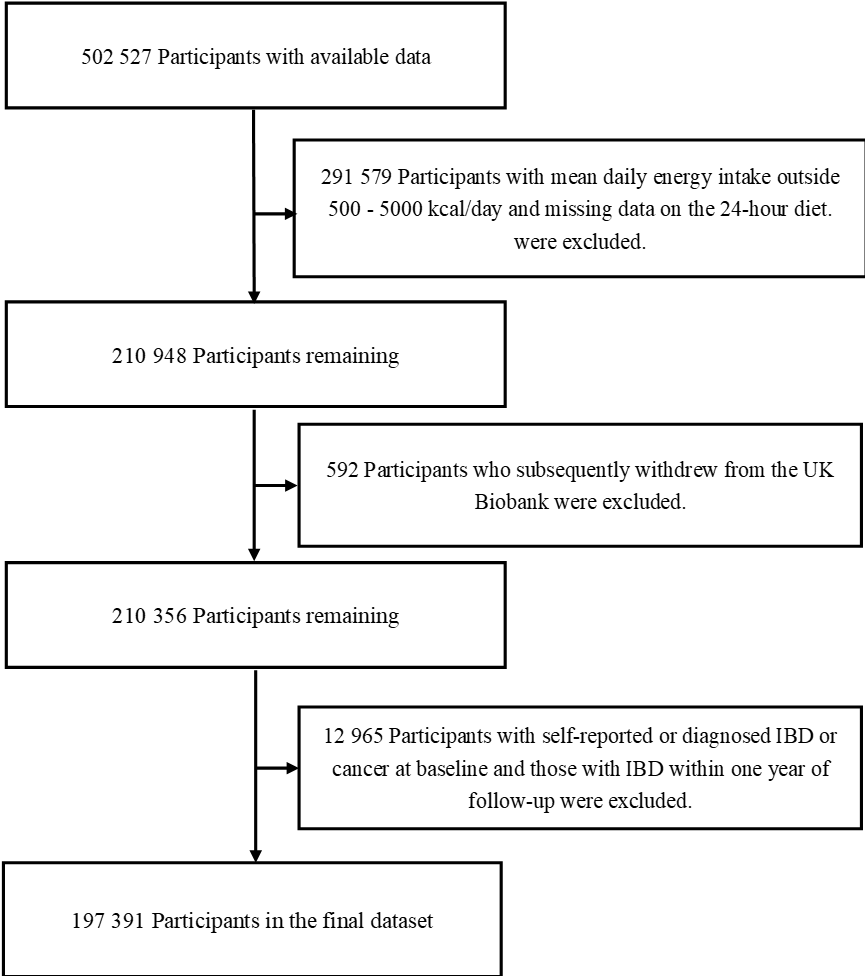
**

**Fig. S1** Flow diagram of participants in the present study.

**Table S1** Components and scoring criteria of the alternate Mediterranean Diet (AMED) score.

| **Components** | **Food** | **Score of 1** | **Score of 0** |
| --- | --- | --- | --- |
| 1.vegetables | coleslaw, side salad, avocado, beetroot, broccoli, butternutsquash, cabbage/kale, carrot, cauliflower, celery, courgette, cucumber, garlic, leek, lettuce, mushroom, onion, parsnip, sweetpepper, spinach, other vegetables | for each component, intakes above the median | for each component, intakes below the median |
| 2.legumes | baked bean, pulses, broad bean,  tofu |  |  |
| 3.fruits | fruit juice, stewed fruit, prune, dried fruit, mixed fruit, apple, banana, berry, cherry, grapefruit, grape, mango, melon, orange, satsuma, other fruit |  |  |
| 4.nuts | salted peanuts, unsalted peanuts, salted nuts, unsalted nuts, seeds |  |  |
| 5.whole grains | muesli, oat crunch, sweetened cereal, plain cereal, bran cereal, whole-wheat cereal, other cereal, oatcakes, whole-meal pasta, brown rice, couscous, cereal bar |  |  |
| 6.fish | tinned tuna, oily fish, breaded fish, battered fish, white fish, prawns, lobster/crab, shellfish, other fish |  |  |
| 7. MUFA: SFA ratio | monounsaturated fatty acids, saturated  fatty acids |  |  |
| 8.red and processed meat | sausage, beef, pork, lamb, bacon, ham, liver | for each component, intakes below the median | for each component, intakes above the median |
| 9.alcohol | alcohol | 5 g/d ≤ alcohol ≤15 g/d | alcohol <5g/d or >15 g/d |
| total score |  | 9 | 0 |

Abbreviations: MUFA, monounsaturated fatty acid; SFA, saturated fatty acid.

**Table S2** Components and scoring criteria of the Healthy Eating Index 2015 (HEI-2015).

| **Components** | **Food** | **Max score** ^a^ | **Criteria for maximum score** | **Score of 0** |
| --- | --- | --- | --- | --- |
| 1.total fruits | stewed fruit, prune, dried fruit, mixed fruit, apple, banana, berry, cherry, grapefruit, grape, mango, melon, orange, satsuma, peach/nectarine, pear, pineapple, plum, other fruit intake | 5 | ≥0.8 cup eq. per 1,000 kcal | no intake |
| 2.whole fruits | fruit juice, stewed fruit, prune, dried fruit, mixed fruit, apple, banana, berry, cherry,  grapefruit, grape, mango, melon, orange, satsuma, peach/nectarine, pear, pineapple, plum, other fruit | 5 | ≥0.4 cup eq. per 1,000 kcal | no intake |
| 3.total vegetables | baked bean, pulses, coleslaw, side salad, avocado, broad bean, green bean, beetroot, broccoli, butternut squash, cabbage/kale, carrot, cauliflower, celery, courgette, cucumber, garlic, leek, lettuce, mushroom, onion, parsnip, pea, sweet pepper, spinach, sprouts, sweetcorn, sweet potato, fresh tomato, tinned tomato, turnip/swede, watercress, other vegetables | 5 | ≥1.1 cup eq. per 1,000 kcal | no intake |
| 4.greens and beans | coleslaw, broccoli, celery, courgette, cucumber, leek, lettuce, spinach, sprouts, watercress, other vegetables, baked bean, pulses, broad bean, green bean, pea | 5 | ≥0.2 cup eq. per 1,000 kcal | no intake |
| 5.whole grains | muesli, oat crunch, sweetened cereal, plain cereal, bran cereal, whole-wheat cereal,  other cereal, oatcakes, whole-meal pasta, brown rice, couscous | 10 | ≥1.5 oz eq. per 1,000 kcal | no intake |
| 6.dairy | milk, flavoured milk, low fat hard cheese, hard cheese, soft cheese, blue cheese, low fat cheese spread, cheese spread, cottage cheese, feta, mozzarella, goats cheese, other cheese, milk-based pudding, other milk-based pudding, yogurt | 10 | ≥1.3 cup eq. per 1,000 kcal | no intake |
| 7.total protein foods | whole egg, scotch egg, other egg, sausage, beef, pork, lamb, crumbed or deep-fried poultry, poultry, bacon, ham, liver, other meat, tinned tuna, oily fish, breaded fish, battered fish, white fish, prawns, lobster/crab, shellfish, other fish, tofu, quorn, other vegetarian alternative, salted peanuts, unsalted peanuts, salted nuts, unsalted nuts, seeds, baked bean, pulses, broad bean, green bean, pea | 5 | ≥2.5 oz eq. per 1,000 kcal | no intake |
| 8.seafood and plant proteins | tinned tuna, oily fish, breaded fish, battered fish, white fish, prawns, lobster/crab, shellfish, other fish, vegetarian sausages/burgers, tofu, quorn, other vegetarian alternative, salted peanuts, unsalted peanuts, salted nuts, unsalted nuts, seeds, baked bean, pulses, broad bean, green bean, pea | 5 | ≥0.8 oz eq. per 1,000 kcal | no intake |
| 9.fatty acids | monounsaturated fatty acids, polyunsaturated fatty acids, saturated fatty acids | 10 | (MUFA +PUFA) /SFA ≥2.5 | (MUFA + PUFA)/SFA≤1.2 |
| 10.refined grains | porridge, sliced bread, baguette, bap, bread roll, naan bread, garlic bread, crispbread, oatcakes, other bread, white pasta, whole-meal pasta, white rice, sushi, snackpot, other grain, double crust pastry, single crust pastry, crumble, pizza, pancake, scotch pancake, cake, doughnut, other dessert | 10 | ≤1.8 oz eq. per 1,000 kcal | ≥4.3 oz eq. per 1,000  kcal |
| 11.sodium | sodium | 10 | ≤1.1 g per 1,000 kcal | ≥2.0 g per 1,000 kcal |
| 12.added sugars | added sugars and preserves | 10 | ≤6.5% of energy | ≥26% of energy |
| 13.saturated fats | saturated fat | 10 | ≤8% of energy | ≥16% of energy |
| total score |  | 100 | | 0 |

Abbreviations: MUFA, monounsaturated fatty acid; PUFA, polyunsaturated fatty acid; SFA, saturated fatty acid; eq., equivalents.

^a^ Intakes between maximum score standard and zero points are scored proportionally.

**Table S3** Components and scoring criteria of the Healthful Plant-based Diet Index (HPDI).

| **Components** | **Food** | **Criteria for maximum score** ^a^ | **Criteria for minimum score** |
| --- | --- | --- | --- |
| 1.whole grains | porridge, muesli, oat crunch, sweetened cereal, plain cereal, bran cereal, whole-wheat cereal, other cereal, whole-meal pasta, brown rice, couscous | for each component, intakes above the highest quintile score 5 | for each component, intakes below the lowest quintile score 1 |
| 2.fruits | avocado, stewed fruit, mixed fruit, apple, banana, berry, cherry, grapefruit, grape, mango, melon, orange, satsuma, peach/nectarine, pear, pineapple, plum, other fruit |  |  |
| 3.vegetables | coleslaw, side salad, beetroot, broccoli, butternut squash, cabbage/kale, carrot,  cauliflower, celery, courgette, cucumber, garlic, leek, lettuce, mushroom, onion, parsnip, pea, sweet pepper, spinach, sprouts, sweetcorn, sweet potato, fresh tomato, tinned tomato, turnip/swede, watercress, other vegetables |  |  |
| 4.nuts | salted peanuts, unsalted peanuts, salted nuts,  unsalted nuts, seeds |  |  |
| 5.legumes | baked bean, pulses, broad bean, green bean, tofu |  |  |
| 6.vegetable oils | Oil-based salad dressing, vegetable oil used for cooking |  |  |
| 7.tea & coffee | instant coffee, filtered coffee, cappuccino, latte, espresso, other coffee type, decaffeinated coffee, standard tea, rooibos tea, green tea, herbal tea, other tea |  |  |
| 8.fruit juices | squash, orange juice, grapefruit juice, pure fruit/vegetable juice | for each component, intakes below the lowest quintile score 5 | for each component, intakes above the highest quintile score 1 |
| 9.refined grains | sliced bread, baguette, bap, bread roll, naan  bread, garlic bread, crispbread, oatcakes, other bread, white pasta, white rice, other grain, pancake, scotch pancake, danish pastry, scone, cereal bar, savoury biscuits, cheesy biscuits |  |  |
| 10.potatoes | fried potatoes, boiled/baked potatoes, mashed potato, crisp |  |  |
| 11.sugar sweetened beverages | fizzy drink, fruit smoothie, dairy smoothie,  other drink |  |  |
| 12.sweets and desserts | hot chocolate, prune, dried fruit, snackpot, double crust pastry, single crust pastry, crumble, milk-based pudding, fruitcake, cake, doughnut, sponge pudding, cheesecake, other dessert, chocolate bar, white chocolate, milk chocolate, dark chocolate, chocolate-covered raisin, chocolate sweet, diet sweets, sweets, chocolate-covered biscuits, chocolate biscuits, sweet biscuits, other sweets, other savoury snack |  |  |
| 13.animal fat | butter/margarine on bread/crackers, butter/margarine added to potatoes |  |  |
| 14.dairy | milk, flavoured milk, yogurt, ice-cream, low  fat hard cheese, hard cheese, soft cheese, blue cheese, low fat cheese spread, cheese spread,  cottage cheese, feta, mozzarella, goats cheese, other cheese |  |  |
| 15.egg | whole egg, omelette, eggs in sandwiches, scotch egg, other egg |  |  |
| 16.fish or seafood | tinned tuna, oily fish, breaded fish, battered fish, white fish, prawns, lobster/crab, shellfish, other fish |  |  |
| 17.meat | sausage, beef, pork, lamb, crumbed or deep-fried poultry, poultry, bacon, ham, liver, other meat |  |  |
| 18. miscellaneous animal-based foods | pizza, chowder or cream soup |  |  |
| total score |  | 90 | 18 |

^a^ Each component is divided into quintiles of consumption, and each quintile is assigned a score of 5 to 1.

**Table S4** Components and scoring criteria of EAT-Lancet Score.

| **Components** | **Food** | **Score for 1 point** |
| --- | --- | --- |
| 1.whole grains | muesli, oat crunch, sweetened cereal, plain cereal, bran cereal, whole-wheat cereal, other cereal, sliced bread, baguette, bap, bread roll, naan bread, garlic bread, crispbread, oatcakes, other bread, cereal bar | ≤ 464 g/day |
| 2.vegetables | vegetable pieces, coleslaw, side salad, avocado, beetroot, broccoli, butternut squash, cabbage/kale, carrot, cauliflower, celery, courgette, cucumber, garlic, leek, lettuce, mushroom, other vegetables | ≥ 200 g/day |
| 3.fruits | squash, orange juice, grapefruit juice, pure fruit/vegetable juice, fruit smoothie, stewed fruit, prune, dried fruit, mixed fruit, apple, banana, berry, cherry, grapefruit, other fruit | ≥ 100 g/day |
| 4.tubers and starchy vegetables | fried potatoes, boiled/baked potatoes, mashed potato, crisp | ≤ 100 g/day |
| 5.dry beans, lentils, peas | baked bean, pulses, broad bean, green bean, pea | ≤100g/day |
| 6.soy foods | vegetarian sausages/burgers, tofu | ≤50g/day |
| 7.peanuts or treenuts | salted peanuts, unsalted peanuts, salted nuts, unsalted nuts, seeds | ≥25g/day |
| 8.dairy foods | low fat hard cheese, hard cheese, soft cheese, blue cheese, low fat cheese spread, cheese spread, cottage cheese, feta, mozzarella, goats cheese, other cheese, milk-based pudding, other milk-based pudding, dairy smoothie, milk, flavoured milk, yogurt | ≤500g/day |
| 9.added sugar | sugar added to coffee, sugar added to tea, sugar added to cereal | ≤31g/day |
| 10.beef, lamb, pork | beef, lamb, pork, bacon, ham | ≤28g/day |
| 11.poultry | crumbed or deep-fried poultry, poultry | ≤58g/day |
| 12.eggs | whole egg, omelette, eggs in sandwiches, scotch egg, other egg | ≤25g/day |
| 13.fish | tinned tuna, oily fish, breaded fish, battered fish, white fish, prawns, lobster/crab, shellfish, other fish | ≤100g/day |
| 14.added fats | monounsaturated fatty acids, polyunsaturated fatty acids, saturated fat | ≥0.8 for unsaturated: saturated fat ratio |
| total score |  | 14 |

**Table S5** Sensitivity analyses of association between healthy eating score and risk of Crohn’s disease and ulcerative colitis. ^a^

|  | **Crohn’s Disease** | | | | |  | **Ulcerative Colitis** | | | |  |
| --- | --- | --- | --- | --- | --- | --- | --- | --- | --- | --- | --- |
| **Variables** | **Sensitivity analysis 1** ^b^ | **Sensitivity analysis 2** ^c^ | **Sensitivity analysis 3** ^d^ | **Sensitivity analysis 4** ^e^ | **Sensitivity analysis 5** ^f^ |  | **Sensitivity analysis 1** ^b^ | **Sensitivity analysis 2** ^c^ | **Sensitivity analysis 3** ^d^ | **Sensitivity analysis 4** ^e^ | **Sensitivity analysis 5** ^f^ |
| **AMED score** |  |  |  |  |  |  |  |  |  |  |  |
| 0-3 | 1.00[Reference] | 1.00[Reference] | 1.00[Reference] | 1.00[Reference] | 1.00[Reference] |  | 1.00[Reference] | 1.00[Reference] | 1.00[Reference] | 1.00[Reference] | 1.00[Reference] |
| 4-5 | 0.89[0.67,1.19] | 0.89[0.67,1.18] | 0.91[0.69,1.2] | 0.88[0.67,1.16] | 0.75 [0.52,1.08] |  | 0.88[0.72,1.08] | 0.92[0.75,1.12] | 0.9[0.74,1.09] | 0.90[0.75,1.09] | 0.87 [0.68,1.11] |
| 6-9 | 0.45[0.28,0.74] | 0.47[0.3,0.75] | 0.48[0.3,0.76] | 0.56[0.37,0.85] | 0.49 [0.28,0.86] |  | 0.87[0.67,1.13] | 0.86[0.67,1.12] | 0.82[0.63,1.06] | 0.88[0.69,1.13] | 0.80 [0.58,1.11] |
| *P* trend | 0.009 | 0.013 | 0.018 | 0.027 | 0.023 |  | 0.348 | 0.300 | 0.136 | 0.212 | 0.117 |
| Per SD increase | 0.83[0.72,0.95] | 0.84[0.73,0.96] | 0.85[0.75,0.97] | 0.86[0.76,0.98] | 0.82 [0.69,0.97] |  | 0.96[0.87,1.05] | 0.95[0.87,1.04] | 0.94[0.86,1.02] | 0.95[0.87,1.03] | 0.91 [0.82,1.02] |
| **HEI-2015** |  |  |  |  |  |  |  |  |  |  |  |
| Q1 | 1.00[Reference] | 1.00[Reference] | 1.00[Reference] | 1.00[Reference] | 1.00[Reference] |  | 1.00[Reference] | 1.00[Reference] | 1.00[Reference] | 1.00[Reference] | 1.00[Reference] |
| Q2 | 0.78[0.57,1.07] | 0.79[0.58,1.08] | 0.82[0.6,1.1] | 0.78[0.58,1.05] | 1.00 [0.68,1.48] |  | 0.99[0.8,1.23] | 1[0.8,1.24] | 1[0.82,1.23] | 1.05[0.87,1.28] | 0.96 [0.73,1.25] |
| Q3 | 0.63[0.44,0.88] | 0.6[0.43,0.84] | 0.64[0.46,0.89] | 0.65[0.47,0.89] | 0.73 [0.47,1.12] |  | 0.87[0.69,1.09] | 0.88[0.7,1.1] | 0.85[0.69,1.06] | 0.88[0.71,1.09] | 0.92 [0.70,1.22] |
| *P* trend | 0.02 | 0.009 | 0.027 | 0.027 | 0.050 |  | 0.234 | 0.171 | 0.105 | 0.140 | 0.392 |
| Per SD increase | 0.85[0.74,0.97] | 0.83[0.72,0.95] | 0.86[0.76,0.98] | 0.87[0.76,0.98] | 0.86 [0.73,1.00] |  | 0.95[0.86,1.04] | 0.94[0.85,1.03] | 0.93[0.85,1.02] | 0.94[0.86,1.02] | 0.95 [0.86,1.06] |
| **HPDI** |  |  |  |  |  |  |  |  |  |  |  |
| Q1 | 1.00[Reference] | 1.00[Reference] | 1.00[Reference] | 1.00[Reference] | 1.00[Reference] |  | 1.00[Reference] | 1.00[Reference] | 1.00[Reference] | 1.00[Reference] | 1.00[Reference] |
| Q2 | 1.09[0.77,1.54] | 1.03[0.73,1.45] | 1.05[0.75,1.46] | 1.05[0.76,1.46] | 1.24 [0.80,1.92] |  | 1.12[0.9,1.41] | 1.08[0.86,1.36] | 1.13[0.91,1.4] | 1.04[0.85,1.29] | 1.29 [0.97,1.70] |
| Q3 | 1.14[0.81,1.61] | 1.22[0.88,1.7] | 1.21[0.88,1.67] | 1.24[0.90,1.69] | 1.42 [0.93,2.17] |  | 1.04[0.83,1.3] | 1.07[0.85,1.34] | 1.07[0.86,1.33] | 1.04[0.84,1.28] | 1.17 [0.88,1.55] |
| *P* trend | 0.66 | 0.979 | 0.894 | 0.991 | 0.792 |  | 0.969 | 0.776 | 0.77 | 0.878 | 0.514 |
| Per SD increase | 0.97[0.85,1.11] | 1[0.88,1.15] | 0.99[0.87,1.13] | 1.00[0.88,1.14] | 1.02 [0.86,1.21] |  | 1[0.91,1.1] | 1.01[0.93,1.11] | 1.01[0.93,1.1] | 1.01[0.93,1.10] | 1.04 [0.93,1.16] |
| **EAT-Lancet score** |  |  |  |  |  |  |  |  |  |  |  |
| Q1 | 1.00[Reference] | 1.00[Reference] | 1.00[Reference] | 1.00[Reference] | 1.00[Reference] |  | 1.00[Reference] | 1.00[Reference] | 1.00[Reference] | 1.00[Reference] | 1.00[Reference] |
| Q2 | 0.82[0.57,1.19] | 0.9[0.62,1.29] | 0.88[0.62,1.25] | 0.91[0.65,1.28] | 0.88 [0.56,1.38] |  | 1.24[0.98,1.57] | 1.16[0.92,1.47] | 1.2[0.96,1.5] | 1.21[0.97,1.50] | 1.39 [1.05,1.86] |
| Q3 | 1.02[0.74,1.4] | 1.14[0.83,1.56] | 1.07[0.79,1.46] | 1.10[0.82,1.49] | 1.04 [0.7,1.55] |  | 1.03[0.82,1.29] | 0.98[0.78,1.22] | 1.01[0.82,1.25] | 1.05[0.85,1.29] | 1.09 [0.82,1.44] |
| *P* trend | 0.625 | 0.917 | 0.803 | 0.962 | 0.941 |  | 0.793 | 0.633 | 0.655 | 0.862 | 0.63 |
| Per SD increase | 0.97[0.84,1.11] | 1.01[0.88,1.15] | 0.98[0.86,1.12] | 1.00[0.88,1.13] | 0.99 [0.84,1.17] |  | 0.99[0.9,1.08] | 0.98[0.89,1.07] | 0.98[0.9,1.07] | 0.99[0.91,1.08] | 1.03 [0.92,1.15] |

Abbreviations: SD, standard deviation; Q, quantile.

^a^ Hazard ratio and 95% CI were estimated from multivariate Cox regression models stratified by sex, age, and assessment center and adjusted for sociodemographic characteristics (ethnicity, index of multiple deprivations, and BMI), lifestyle factor (smoking status, alcohol consumption, physical activity, and daily sleeping time), medications (multivitamins, mineral, aspirin, non-aspirin NSAIDs, and statins use), comorbidities (hypercholesterolemia, hypertension, diabetes, and longstanding illness).

^b^ Sensitivity analysis 1 lagged the exposure by 2 years.

^c^ Sensitivity analysis 2 excluded participants who reported their atypical diet.

^d^ Sensitivity analysis 3 removed the variable BMI from the multivariable-adjusted model.

^e^ Sensitive analysis 4 excluded participants with digestive system diseases at baseline and those who developed digestive system diseases during the period from baseline to the dietary questionnaire survey.

^f^ Sensitive analysis 5 excluded participants with a time gap of more than two years between covariate and dietary data collection.

**Table S6** Subgroup analyses of association between per SD increase of healthy eating score and risk of Crohn’s disease. ^a^

| **Variables** | **AMED** | ***P* interaction** |  | **HEI-2015** | ***P* interaction** |  | **HPDI** | ***P* interaction** |  | **EAT-Lancet** | ***P* interaction** |
| --- | --- | --- | --- | --- | --- | --- | --- | --- | --- | --- | --- |
| **Sex** |  |  |  |  |  |  |  |  |  |  |  |
| Male | 0.93[0.76,1.12] | 0.285 |  | 0.90[0.74,1.10] | 0.584 |  | 0.84[0.69,1.01] | 0.014 |  | 0.87[0.72,1.05] | 0.063 |
| Female | 0.80[0.67,0.97] |  |  | 0.84[0.7,1.00] |  |  | 1.18[0.99,1.42] |  |  | 1.13[0.94,1.36] |  |
| **Age** |  |  |  |  |  |  |  |  |  |  |  |
| < 60 | 0.83[0.69,0.99] | 0.412 |  | 0.95[0.80,1.13] | 0.153 |  | 1.06[0.90,1.26] | 0.288 |  | 1.04[0.88,1.23] | 0.513 |
| ≥ 60 | 0.89[0.73,1.09] |  |  | 0.75[0.61,0.92] |  |  | 0.90[0.73,1.09] |  |  | 0.92[0.75,1.12] |  |
| **Obesity** |  |  |  |  |  |  |  |  |  |  |  |
| No | 0.84[0.66,1.06] | 0.873 |  | 0.72[0.57,0.91] | 0.104 |  | 0.95[0.75,1.19] | 0.569 |  | 1.06[0.84,1.33] | 0.597 |
| Yes | 0.86[0.73,1.01] |  |  | 0.94[0.80,1.10] |  |  | 1.03[0.88,1.2] |  |  | 0.96[0.82,1.13] |  |
| **Never smoker** |  |  |  |  |  |  |  |  |  |  |  |
| Yes | 0.91[0.76,1.10] | 0.310 |  | 0.89[0.74,1.07] | 0.545 |  | 0.98[0.82,1.18] | 0.954 |  | 0.99[0.83,1.2] | 0.904 |
| No | 0.82[0.68,0.99] |  |  | 0.85[0.71,1.03] |  |  | 1.02[0.85,1.23] |  |  | 0.98[0.82,1.18] |  |
| **Never drinker** |  |  |  |  |  |  |  |  |  |  |  |
| Yes | 0.69[0.52,0.93] | 0.073 |  | 0.74[0.57,0.97] | 0.216 |  | 1.16[0.90,1.50] | 0.196 |  | 1.17[0.90,1.53] | 0.123 |
| No | 0.92[0.79,1.07] |  |  | 0.91[0.78,1.06] |  |  | 0.96[0.82,1.11] |  |  | 0.94[0.81,1.09] |  |
| **Physical activity** | |  |  |  |  |  |  |  |  |  |  |
| < Median | 0.90[0.76,1.07] | 0.462 |  | 0.95[0.80,1.12] | 0.155 |  | 0.97[0.82,1.15] | 0.605 |  | 0.91[0.76,1.07] | 0.145 |
| ≥ Median | 0.82[0.66,1.00] |  |  | 0.78[0.63,0.96] |  |  | 1.06[0.87,1.30] |  |  | 1.12[0.91,1.37] |  |
| **Regular NSAIDs use** | |  |  |  |  |  |  |  |  |  |  |
| No | 0.86[0.75,1.00] | 0.688 |  | 0.86[0.75,1.00] | 0.899 |  | 1.05[0.91,1.21] | 0.170 |  | 0.97[0.84,1.11] | 0.493 |
| Yes | 0.82[0.59,1.12] |  |  | 0.87[0.63,1.20] |  |  | 0.82[0.60,1.12] |  |  | 1.15[0.84,1.56] |  |

Abbreviation: NSAIDs, non-steroidal anti-inflammatory drugs.

^a^ Estimated effects were based on the fully adjusted model (see footnote in table S5).

**Table S7** Subgroup analyses of association between per SD increase of healthy eating score and risk of Ulcerative colitis.

| **Variables** | **AMED** | ***P* interaction** |  | **HEI-2015** | ***P* interaction** |  | **HPDI** | ***P* interaction** |  | **EAT-Lancet** | ***P* interaction** |
| --- | --- | --- | --- | --- | --- | --- | --- | --- | --- | --- | --- |
| **Sex** |  |  |  |  |  |  |  |  |  |  |  |
| Male | 0.93[0.82,1.06] | 0.903 |  | 0.86[0.76,0.98] | 0.081 |  | 1.00[0.88,1.13] | 0.679 |  | 1.00[0.88,1.13] | 0.832 |
| Female | 0.95[0.84,1.07] |  |  | 1.01[0.89,1.14] |  |  | 1.04[0.92,1.18] |  |  | 0.97[0.86,1.10] |  |
| **Age** |  |  |  |  |  |  |  |  |  |  |  |
| < 60 | 0.90[0.80,1.02] | 0.409 |  | 0.93[0.83,1.04] | 0.942 |  | 1.01[0.90,1.13] | 0.937 |  | 1.03[0.92,1.15] | 0.175 |
| ≥ 60 | 0.98[0.86,1.11] |  |  | 0.92[0.81,1.05] |  |  | 1.00[0.88,1.15] |  |  | 0.92[0.80,1.05] |  |
| **Obesity** |  |  |  |  |  |  |  |  |  |  |  |
| No | 0.99[0.85,1.16] | 0.580 |  | 0.97[0.83,1.14] | 0.832 |  | 1.07[0.91,1.26] | 0.758 |  | 1.05[0.89,1.23] | 0.555 |
| Yes | 0.92[0.83,1.02] |  |  | 0.92[0.82,1.02] |  |  | 1.00[0.90,1.11] |  |  | 0.97[0.87,1.07] |  |
| **Never smoker** |  |  |  |  |  |  |  |  |  |  |  |
| Yes | 0.94[0.82,1.07] | 0.988 |  | 0.90[0.79,1.03] | 0.421 |  | 0.97[0.85,1.11] | 0.501 |  | 0.98[0.86,1.12] | 0.886 |
| No | 0.93[0.83,1.05] |  |  | 0.96[0.85,1.08] |  |  | 1.05[0.94,1.18] |  |  | 0.99[0.88,1.11] |  |
| **Never drinker** |  |  |  |  |  |  |  |  |  |  |  |
| Yes | 0.95[0.77,1.16] | 0.791 |  | 0.91[0.75,1.10] | 0.906 |  | 0.98[0.81,1.19] | 0.792 |  | 1.02[0.84,1.24] | 0.553 |
| No | 0.94[0.85,1.03] |  |  | 0.94[0.85,1.04] |  |  | 1.03[0.93,1.13] |  |  | 0.98[0.89,1.08] |  |
| **Physical activity** | |  |  |  |  |  |  |  |  |  |  |
| < Median | 0.90[0.80,1.01] | 0.208 |  | 0.91[0.81,1.02] | 0.510 |  | 0.99[0.88,1.11] | 0.719 |  | 0.94[0.84,1.05] | 0.323 |
| ≥Median | 0.99[0.86,1.13] |  |  | 0.96[0.84,1.09] |  |  | 1.04[0.91,1.19] |  |  | 1.04[0.92,1.19] |  |
| **Regular NSAIDs use** | |  |  |  |  |  |  |  |  |  |  |
| No | 0.92[0.83,1.01] | 0.327 |  | 0.93[0.84,1.02] | 0.904 |  | 1.00[0.91,1.10] | 0.542 |  | 0.96[0.87,1.05] | 0.184 |
| Yes | 1.05[0.84,1.31] |  |  | 0.96[0.77,1.20] |  |  | 1.13[0.91,1.41] |  |  | 1.16[0.93,1.44] |  |

Abbreviation: NSAIDs, non-steroidal anti-inflammatory drugs.

^a^ Estimated effects were based on the fully adjusted model (see footnote in table S5).

**Table S8** Associations of per SD score of healthy eating patterns with inflammation biomarkers.

| **Variables** | **β [95% CI]** ^a^ | ***P* value** |
| --- | --- | --- |
| **INFLA-score** |  |  |
| AMED score | -0.231[-0.260,-0.202] | ＜0.001 |
| HEI-2015 | -0.273[-0.302,-0.244] | ＜0.001 |
| **WBC** |  |  |
| AMED score | -0.042[-0.050,-0.033] | ＜0.001 |
| HEI-2015 | -0.048[-0.056,-0.039] | ＜0.001 |
| **PLT** |  |  |
| AMED score | -1.448[-1.733,-1.163] | ＜0.001 |
| HEI-2015 | -1.963[-2.249,-1.677] | ＜0.001 |
| **CRP** |  |  |
| AMED score | -0.071[-0.090,-0.052] | ＜0.001 |
| HEI-2015 | -0.059[-0.079,-0.040] | ＜0.001 |
| **NLR** |  |  |
| AMED score | -0.006[-0.012,-0.001] | ＜0.001 |
| HEI-2015 | -0.012[-0.018,-0.007] | ＜0.001 |

Abbreviations: CI, confidence interval; SD, standard deviation; INFLA-score, low-grade inflammation score; WBC, white blood cell; PLT, platelet; CRP, C-reactive protein; NLR, neutrophil-to-lymphocyte ratio.

^a^ Regression coefficient beta and 95% CI were estimated from multivariate-linear regression models stratified by sex, age, and assessment center and adjusted for sociodemographic characteristics (ethnicity, index of multiple deprivations, and BMI), lifestyle factor (smoking status, alcohol consumption, physical activity, and sleep quality), medication (multivitamins, mineral, aspirin, non-aspirin NSAIDs, and statins use), baseline disease (hypercholesterolemia, hypertension, diabetes, and longstanding illness).

**Table S9** Association between Inflammatory Factors and the Risk of Crohn’s disease and ulcerative colitis.

| **Variables** | **Crohn’s disease** | |  | **Ulcerative colitis** | |
| --- | --- | --- | --- | --- | --- |
|  | **Age and gender-stratified HR [95% CI]** | **Multivariable-adjusted HR [95% CI]** ^a^ |  | **Age and gender-stratified HR [95% CI]** | **Multivariable-adjusted HR [95% CI]** ^a^ |
| **INFLA-score** | | | | | |
| Q1 | 1.00[Reference] | 1.00[Reference] |  | 1.00[Reference] | 1.00[Reference] |
| Q2 | 1.15[0.70,1.90] | 1.13[0.68,1.86] |  | 0.98[0.68,1.41] | 1.00[0.70,1.45] |
| Q3 | 1.47[0.93,2.33] | 1.35[0.85,2.16] |  | 1.90[1.40,2.58] | 1.84[1.34,2.52] |
| Q4 | 2.42[1.57,3.74] | 2.06[1.31,3.26] |  | 2.45[1.81,3.31] | 2.28[1.65,3.14] |
| *P* trend | ＜0.001 | ＜0.001 |  | ＜0.001 | ＜0.001 |
| **WBC** | | | | | |
| Q1 | 1.00[Reference] | 1.00[Reference] |  | 1.00[Reference] | 1.00[Reference] |
| Q2 | 1.46[0.92,2.31] | 1.39[0.87,2.21] |  | 1.09[0.80,1.50] | 1.06[0.77,1.47] |
| Q3 | 1.63[1.04,2.57] | 1.56[0.99,2.48] |  | 1.49[1.11,2.01] | 1.46[1.08,1.98] |
| Q4 | 2.18[1.42,3.35] | 1.91[1.22,3.00] |  | 2.03[1.54,2.68] | 1.88[1.40,2.53] |
| *P* trend | ＜0.001 | 0.001 |  | ＜0.001 | ＜0.001 |
| **PLT** | | | | | |
| Q1 | 1.00[Reference] | 1.00[Reference] |  | 1.00[Reference] | 1.00[Reference] |
| Q2 | 1.15[0.76,1.74] | 1.16[0.76,1.77] |  | 0.92[0.68,1.23] | 0.94[0.70,1.27] |
| Q3 | 1.11[0.73,1.71] | 1.09[0.70,1.68] |  | 1.43[1.09,1.87] | 1.45[1.10,1.90] |
| Q4 | 1.55[1.04,2.33] | 1.45[0.95,2.19] |  | 1.20[0.90,1.59] | 1.16[0.87,1.56] |
| *P* trend | 0.115 | 0.230 |  | 0.101 | 0.182 |
| **CRP** | | | | | |
| Q1 | 1.00[Reference] | 1.00[Reference] |  | 1.00[Reference] | 1.00[Reference] |
| Q2 | 1.12[0.68,1.84] | 1.10[0.67,1.82] |  | 1.40[1.02,1.91] | 1.43[1.04,1.97] |
| Q3 | 1.58[0.99,2.51] | 1.53[0.95,2.46] |  | 1.63[1.21,2.22] | 1.64[1.19,2.25] |
| Q4 | 2.75[1.80,4.21] | 2.41[1.51,3.86] |  | 2.11[1.57,2.82] | 1.90[1.36,2.63] |
| *P* trend | ＜0.001 | ＜0.001 |  | ＜0.001 | ＜0.001 |
| **NLR** | | | | | |
| Q1 | 1.00[Reference] | 1.00[Reference] |  | 1.00[Reference] | 1.00[Reference] |
| Q2 | 1.31[0.85,2.00] | 1.34[0.87,2.05] |  | 1.33[0.98,1.81] | 1.36[1.00,1.85] |
| Q3 | 1.03[0.66,1.62] | 1.02[0.65,1.61] |  | 1.63[1.21,2.18] | 1.62[1.20,2.19] |
| Q4 | 1.76[1.18,2.64] | 1.68[1.11,2.54] |  | 1.84[1.38,2.45] | 1.81[1.35,2.43] |
| *P* trend | ＜0.001 | 0.001 |  | ＜0.001 | ＜0.001 |

Abbreviations: HR, hazard ratio; CI, confidence interval; Q, quantile; INFLA-score, low-grade inflammation score; WBC, white blood cell; PLT, platelet; CRP, C-reactive protein; NLR, neutrophil-to-lymphocyte ratio.

^a^ Estimated effects were based on the fully adjusted model (see footnote in table S5).
